# Battling Chemoresistance in Cancer: Root Causes and Strategies to Uproot Them

**DOI:** 10.3390/ijms22179451

**Published:** 2021-08-31

**Authors:** Alisha Ramos, Samira Sadeghi, Hossein Tabatabaeian

**Affiliations:** 1 Department of Biochemistry, Yong Loo Lin School of Medicine, National University of Singapore, Singapore 117545, Singapore; a0123642@u.nus.edu; 2 Department of Medicine, Yong Loo Lin School of Medicine, National University of Singapore, Singapore 119228, Singapore; samira.sadeghi@u.nus.edu; 3 Genome Institute of Singapore (GIS), Agency for Science, Technology and Research (A*STAR), Singapore 138672, Singapore; 4 Cancer Science Institute of Singapore, National University of Singapore, Singapore 117599, Singapore

**Keywords:** cancer, cancer therapy, chemoresistance

## Abstract

With nearly 10 million deaths, cancer is the leading cause of mortality worldwide. Along with major key parameters that control cancer treatment management, such as diagnosis, resistance to the classical and new chemotherapeutic reagents continues to be a significant problem. Intrinsic or acquired chemoresistance leads to cancer recurrence in many cases that eventually causes failure in the successful treatment and death of cancer patients. Various determinants, including tumor heterogeneity and tumor microenvironment, could cause chemoresistance through a diverse range of mechanisms. In this review, we summarize the key determinants and the underlying mechanisms by which chemoresistance appears. We then describe which strategies have been implemented and studied to combat such a lethal phenomenon in the management of cancer treatment, with emphasis on the need to improve the early diagnosis of cancer complemented by combination therapy.

## 1. Cancer and Chemoresistance

**Figure 1:**
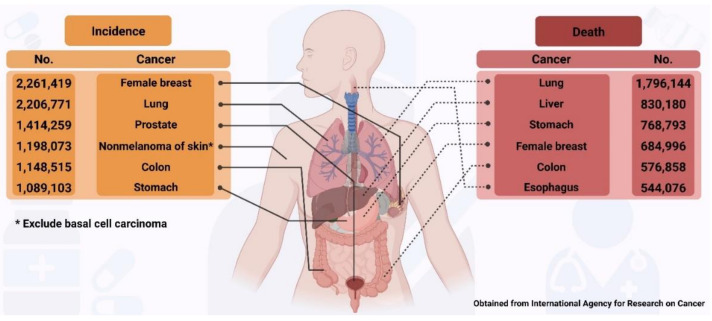
Incidence and mortality of cancers reported in 2020 globally.

Cancer is one of the major causes of death globally, accounting for 10 million deaths in 2020 [1]. The most common cancers and those responsible for deaths reported in 2020 in both men and women are shown in Figure 1, which highlights a major concern regarding the number of humans affected by cancer worldwide.

**Figure 2:**
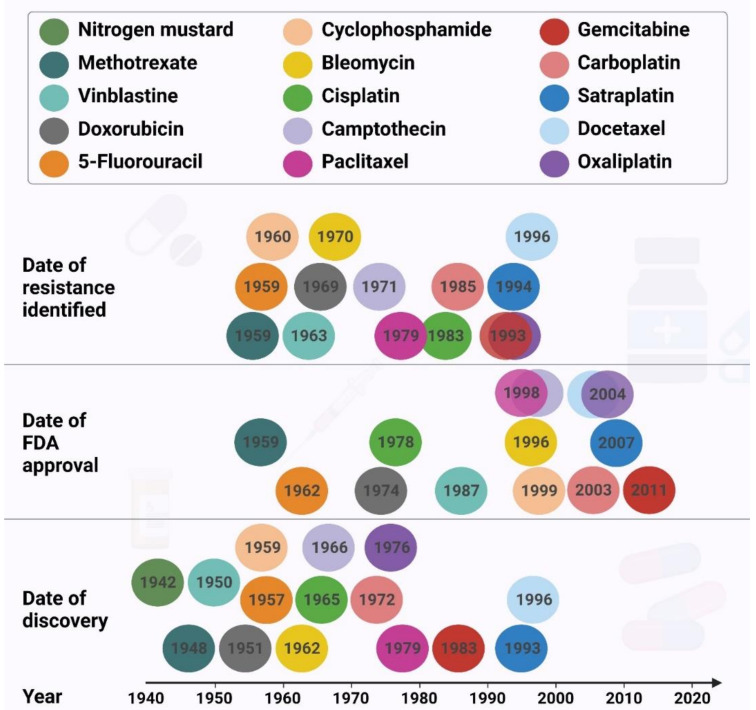
The timelines of chemotherapeutic agent discovery, date of approval by the Food and Drug Association of the United States of America, and the approximate date the resistance was identified.

Surgery, hormone therapy, gene therapy, immunotherapy, radiation therapy, laser therapy, combination therapy, and targeted therapy are the major cancer treatments available, with chemotherapy being the most common and promising treatment for cancer management [2,3,4]. Despite the progress made in cancer treatment, resistance to chemotherapeutic drugs continues to be a major problem in cancer therapy (Figure 2), and is responsible for most relapses and poor survival outcomes in patients.

The resistance to the chemotherapeutic regimens has been reported for almost all the drugs used to treat the most lethal cancers. The resistance to doxorubicin [5], paclitaxel [6,7,8], 5-fluorouracil [9], cyclophosphamide [10,11] and carboplatin [12] have been shown to cause cancer recurrence in breast cancer associated with poorer prognosis and shorter survival. Resistance to 5-fluorouracil [13], cisplatin [14,15,16], docetaxel [17,18] and oxaliplatin [19] result in the same outcomes in gastric cancer patients. Moreover, the resistance to 5-fluorouracil [20], irinotecan [21], and oxaliplatin [22] in colorectal cancer, cisplatin [23,24], carboplatin [25,26], paclitaxel 23] and docetaxel [27,28] in lung, and gemcitabine [29], oxaliplatin [30], cisplatin [28,31] and doxorubicin [28] strongly indicate that the chemoresistance phenomenon intensively threatens the health and survival of cancer patients. Approximately 80–90% of mortality in cancer patients is directly or indirectly attributed to drug resistance [32]. Resistance can be restricted to a specific drug, or different drugs with independent modes of action, named multidrug resistance (MDR).

## 2. Types and Determinants of Chemoresistance

**Figure 3:**
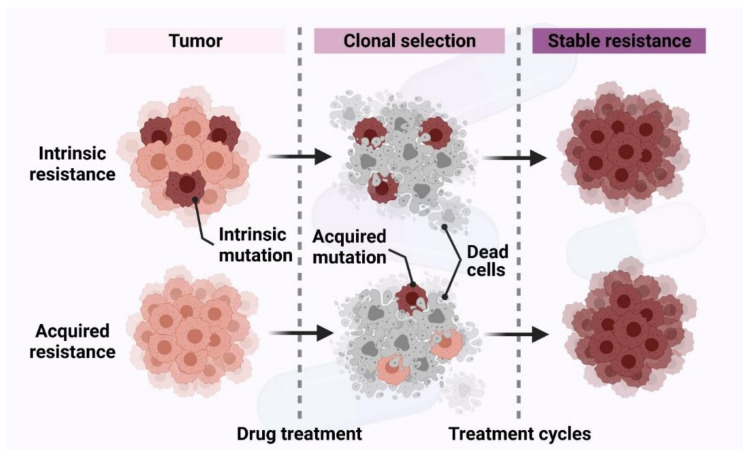
Intrinsic and acquired chemoresistance in cancer.

Chemoresistance (drug resistance) is classified into two categories—intrinsic and acquired resistance depending upon its time of development (Figure 3). Early diagnosis of the types of drug resistance helps pre-determine the sensitivity of the cancer cells to the drug, optimize therapy and reduce the toxic side effects [33].

### 2.1. Intrinsic Chemoresistance

Intrinsic chemoresistance exists before the drug/therapy has been administered to the patient (Figure 3). Tumors with intrinsic resistance show a resistant phenotype to chemotherapy before they encounter any chemotherapeutic drugs [34]. The determinants of this resistance are heterogeneous and can be listed as follows.

#### 2.1.1. Inherent Genetic Mutations in Tumors

The Kelch-like ECH-associated protein 1 (KEAP1)-Nuclear factor (erythroid-derived 2)-like 2 (NFE2L2) pathway regulates redox and metabolic homeostasis. NFE2L2 is a transcription factor that regulates the transcription of antioxidants, growth factors, detoxifying genes, and drug efflux genes. NFE2L2 activity is regulated by KEAP1, a cytoplasmic adaptor protein of the Cullin3 (Cul3)-based E3-ligase. Mutations in these two genes affecting their interaction or resulting in NFE2L2 overexpression have been identified in several cancer tissues, such as lung, breast, bladder, ovarian and liver [35]. Genes in the KEAP1–NFE2L2 pathway are mutated in 33% of lung squamous cell carcinoma (LSCC) [36,37] and 22% of lung adenocarcinoma (LUAD) [38]. Animal studies in non-small cell lung cancer (NSCLC) show that KEAP1 deletion and NFE2L2 mutations promote cancer cell growth and a pro-survival phenotype in these cells. *KEAP1* deletion confers chemoresistance in preclinical models of LUAD and LSCC. NSCLC patients with *KEAP1*/*NFE2L2*/*CUL3* mutations have significantly shorter time to treatment failure (TTF) and overall survival (OS) when treated with first-line chemotherapy [39].

#### 2.1.2. Heterogeneity of Tumor Cell Population

Cancer stem cells (CSCs) play a crucial role in intrinsic chemoresistance in different cancer types. CSCs constitute a smaller sub-population as compared to terminally differentiated cancer cells. Post-chemotherapy, this sub-population of cells is enriched in many different cancers [40,41,42]. For instance, the breast CSCs (BCSCs) display properties like self-renewal and differentiation, quiescence, altered metabolism, overexpression of drug efflux transporters, enhanced DNA repair and elude the immune system. These properties support the invasion, metastasis and relapse of cancer in patients post-chemotherapy, ultimately being responsible for the poor clinical outcomes, higher death rates and chemoresistance in breast cancer patients [43,44].

#### 2.1.3. Activation of Intrinsic Pathways

Plenty of intrinsic signaling pathways play pivotal roles in promoting drug resistance in human cancers [45]. For example, activation of intrinsic pathways like mitogen-activated protein kinase (MAPK), hedgehog, phosphoinositide 3-kinase (PI_3_K)/Akt, Nuclear factor-κB (NF_k_B), and notch pathways are responsible for gemcitabine resistance in pancreatic cancer. Activation of these pathways influences angiogenesis, cellular proliferation, concentration and distribution of the drug at the tumor site, apoptosis, and survival, leading to chemoresistance in these patients [46].

#### 2.1.4. Pharmacological Factors

Inadequate intracellular drug concentration at the tumor site, inability of the drug to reach its optimum pharmacokinetic profile, altered drug target, altered absorption, distribution, metabolism, or excretion of a drug can contribute to clinical resistance [47]. Anthracyclins and taxanes are used for the treatment of breast cancer. To carry out their action they need to bind to their targets topoisomerase II (topo-II) and β-tubulin. Anthracyclins bind to topo-II and induce apoptosis in breast cancer cells while taxanes bind to β-tubulin, promoting mitotic arrest and apoptosis. Expression and localization of these targets significantly affect drug sensitivity. ATP-binding cassette (ABC) transporter proteins like multidrug resistance-associated protein 1 (MRP1) and lung resistance-related protein (LRP) are responsible for the efflux of anthracyclins and taxanes from their site of action, resulting in low intracellular concentration of the drug and drug resistance [48].

### 2.2. Acquired Chemoresistance

Acquired drug resistance occurs after chemotherapy treatment. It is identified by the appearance of drug-resistant cell populations and reduced efficacy of the anticancer treatment/drug (Figure 3). The reasons for this type of resistance are mutations of drug targets, activation of the second proto-oncogene, changes in the tumor microenvironment, epigenetic alterations by methylation, acetylation, and microRNA (miRNA) expression leading to alterations in upstream or downstream regulators, alterations in the cell cycle and its checkpoints, impairment of apoptosis, and altered DNA repair [4,32]. Epigenetic alterations are one of the mechanisms of drug resistance in cancer therapy. They can be classified into three categories—1. DNA methylation 2. histone modifications 3. miRNA. These alter the expression of genes, activate oncogenes, and inhibit tumor suppressor genes, leading to chemoresistance. Platinum-based anticancer drugs cisplatin and carboplatin are commonly used to treat ovarian cancer. Epigenetic modifications lead to cis- or carboplatin-resistant ovarian cancers. Hypermethylation-mediated repression of cell adhesion and tight junction pathways as well as hypomethylation-mediated activation of the cell growth-promoting pathways PI_3_K/Akt and Transforming growth factor β (TGF-β) may contribute to platinum resistance [49]. Besides chromosomal modifications, non-coding RNAs such as miRNAs and long non-coding RNAs (lncRNAs) also play an important role in chemoresistance. miRNA contains approximately 22 nucleotides and is a small single-stranded non-coding RNA molecule that functions in RNA silencing and post-transcriptional regulation of gene expression [50,51]. lncRNAs are approximately 200 to more than 10,000 nucleotides in length. lncRNAs regulate gene expression at the transcriptional and post transcriptional level [52,53]. The dysregulation of these non-coding RNA species not only plays a role in promoting cancer [54,55,56,57,58], but also alters the expression of proteins related to cancer drug resistance [59]. For instance, overexpression of miR-499a significantly enhanced the proliferation, cell cycle progression, colony formation, apoptosis resistance, migration and invasion of cervical cancer cells. miR-499a down-regulated SRY-box transcription factor 6 (SOX6), resulting in chemoresistance in cervical cancer calls [60]. lncRNA urothelial cancer-associated 1 (UCA1) was shown to be upregulated in cisplatin-resistant bladder cancer cells compared to sensitive cells. Up-regulation of UCA1 expression resulted in significantly increased mRNA and protein levels of Wnt family member 6 (Wnt6), promoting Wnt signaling and cell survival [61].

Acquired chemoresistance due to changes in gene expression has been observed in hepatocellular carcinoma cells (HCC). In the drug-resistant cells, the G-actin monomer-binding protein thymosin β4 (Tβ4) was enriched through demethylation of DNA and active modification of histone H3 at the promoter region. Overexpression of Tβ4 led to the acquisition of stemness in the HCC cell line and induced resistance to sorafenib a vascular endothelial growth factor receptor (VEGFR) inhibitor in vivo [62].

Intrinsic and acquired resistance can occur concurrently during tumor advancement and treatment. Mechanisms of acquired drug resistance can be totally different from the existing intrinsic drug resistance or can be an expansion of the intrinsic drug resistance. While this section touches upon the different types of chemoresistance, a more detailed explanation of them is provided under the mechanisms of chemoresistance section. Understanding the mechanisms of chemoresistance will aid in developing strategies to overcome them and also in optimizing means to diagnose them at the earliest point.

## 3. Mechanisms of Chemoresistance in Cancer

A chemotherapeutic drug exerts its action in four stages. First, the drug enters the cell, where it is then activated. The drug exerts its effect on its target(s) inside the cell, which subsequently results in cell death. Chemoresistance can come into effect at any of these four stages. Along with the determinants reviewed in the previous section, which resulted in intrinsic or acquired chemoresistance, various mechanisms by which chemoresistance operates are discussed in more detail here.

### 3.1. Tumor Microenvironment (TME)

**Figure 4:**
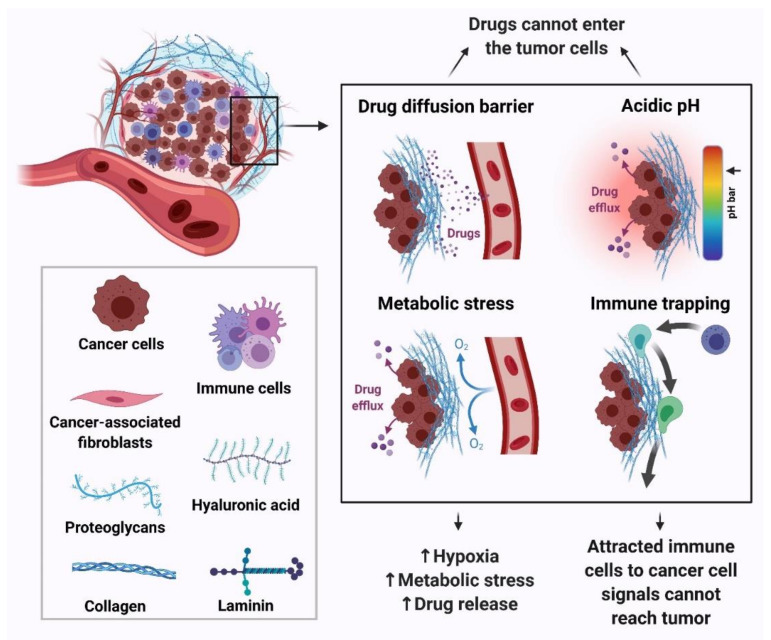
The effect of tumor microenvironment on chemoresistance in human cancers.

The TME comprises tumor cells and stromal cells including stromal fibroblasts, endothelial cells and immune cells surrounded by non-cellular components of the extracellular matrix such as collagen, fibronectin, hyaluronan, and laminin supported by a vascular network. These components work in a coordinated manner and the outcome of this crosstalk results in tumor formation and maintenance as well as poor response to therapy and chemoresistance [63] (Figure 4). The various components of the tumor microenvironment that contribute to drug resistance are hypoxia, reduced glucose supply, vascular viscosity and resistance, co-resistance and diffusion. Cancer cells are more sensitive to glucose concentration changes than normal cells, dying significantly faster than normal cells under glucose deprivation. However, these cells could reprogram the glucose and other nutrient metabolisms to maintain the oncogenic pathways under metabolic stress. Chemo-resistance signaling pathways are deregulated upon metabolic stress, and the key ones are the PI_3_K/Akt, MAPK and Wnt pathways [64,65,66,67,68,69,70]. Moreover, the metabolic reprogramming mediates aerobic glycolysis and the pentose phosphate pathway, eventually resulting in promoting DNA repair and apoptosis resistance [71,72,73].

Drugs pass through blood vessels and then enter the tumor tissue by convection or diffusion to exert their function. Convection is a process dependent on a pressure gradient between the intravascular and interstitial space, vascular permeability and exchange levels, and the volume and structure of the extracellular matrix. In tumors, the oncotic pressure gradient is usually zero, while interstitial fluid pressure is mostly high and equal to capillary blood pressure. This situation decreases macromolecule leakage to the tumor tissue, especially the central part of it, and is considered as a drug resistance method [74]. The interaction of cancer cells with each other and surrounding cytokines, hormones, growth factors and extracellular matrix affect their sensitivity to apoptosis and chemotherapy. This phenomenon is called adhesion-mediated drug resistance or co-resistance. The interaction of very late antigen 4 (VLA-4), vascular adhesion molecule (VCAM), leukocyte function-associated antigen 1 (LFA-1), and intercellular adhesion molecule 1 (ICAM-1) with bone marrow stromal cells induces primary multidrug resistance in vitro and in vivo in multiple myeloma cells [75].

### 3.2. Drug Influx and Efflux

The effectiveness of chemotherapeutic drugs depends on their successful entry into the cells at an optimum intracellular concentration. Drug influx is affected by the location of the tumor within the body, size of the tumor, physics of the tumor site, the structure and function of the tumor vasculature, necrosis of the tumor, transport properties of the drug as it moves through microvessel walls and in the extravascular tissue, alterations in binding properties and number of drug uptake transport systems (influx pumps), mode of drug diffusion, and absorption of the drug and intracellular pH [32,76].

Tumor cells have an acidic pH due to high aerobic glycolysis while anti-cancer drugs are mostly weak bases (daunorubicin, doxorubicin, mitoxantrone) or weak acids (cyclophosphamide, chlorambucil, cisplatin). The weak bases tend to accumulate in the interstitial fluid, and if they cross the plasma membrane they accumulate in acidic membrane compartments, such as the trans-Golgi network, endosomes and lysosomes, thereby decreasing the amount of free drug available to bind to intracellular targets while the weak acids undergo ion trapping in the cytosol and are prevented from reaching their targets [47]. Thus, the acidic pH of tumor cells could result in promoting chemoresistance through decreased drug influx at the site of action (Figure 4).

Solute carrier (SLC) transporters are a family of membrane-bound proteins that serve as influx pumps transporting substrates across biological membranes. They play important roles in biological processes ranging from the cellular uptake of nutrients/vitamins to the absorption of drugs and other xenobiotics. Reduced influx of drugs into the cell can occur in two ways—1. reduced binding of the drugs to the transporters, 2. reducing the numbers of transporters. The glycoprotein-reduced folate carrier (RFC) is an SLC transporter that helps transport the drugs pralatrexate and methotrexate across the cell membrane. Point mutation of G at nucleotide 133 and the substitution of lysine to glutamic acid in the first transmembrane domain of the hRFC protein reduces the tendency of the drug methotrexate to bind the transporter in ALL patients. A decrease in RFC expression leads to methotrexate resistance and poor response to therapy in osteosarcoma and resistance to pralatrexate in multiple myeloma [77,78,79].

In addition, increased efflux prevents the drug from reaching its therapeutically effective concentration. ABC proteins present in the cell membrane are responsible for the regulation of distribution, absorption and excretion of a variety of chemical compounds. These proteins protect cancer cells by expelling drugs from the cell, decreasing the bioavailability of the drugs and maintaining their intracellular concentration below the toxic level. These proteins are characterized by the presence of two distinct domains—a highly conserved nucleotide-binding domain and a more variable transmembrane domain. When a given substrate binds to the transmembrane domain, ATP hydrolysis at the nucleotide-binding site drives a change in conformation that pushes the substrate out of the cell. While efflux via ABC transporters is a normal physiological process, it is also a known mechanism of drug resistance in cancer cells. These proteins are overexpressed in cancers and function to efflux drugs from the cell, leading to chemoresistance (Table 1). P-glycoprotein and ATP-binding cassette G2 transporter (ABCG2) preferentially extrude large, hydrophobic, positively charged molecules, while the members of the MRP family can extrude both hydrophobic uncharged molecules and water-soluble anionic compounds [34,74,76,80,81,82,83,84,85].

### 3.3. Epithelial-Mesenchymal Transition (EMT)

EMT is the process by which epithelial cells acquire a mesenchymal phenotype. They lose cell polarity and cell-to-cell adhesion and gain migratory and invasive properties, thus promoting metastasis. EMT is related to cancer progression, metastasis and mediates drug resistance to chemotherapy. Several factors like reduced expression of epithelial markers E-cadherin and occludin and increased expression of mesenchymal markers vimentin, fibronectin and N-cadherin, tumor microenvironment, hypoxia and signal transduction pathways (WNT, Notch, Hedgehog) can trigger EMT, leading to drug resistance [86]. EMT-inducing transcriptional factors (EMT-TFs) play a role in drug resistance. Overexpression of EMT-TFs like Twist, Snail, Slug, Zinc finger E-box binding homeobox 1 (ZEB1) and Forkhead box C2 (FOXC2) are known to induce drug resistance in breast cancer [87]. ZEB1 induces EMT by suppressing the epithelial phenotype by repressing epithelial microRNAs such as miRNA-200 family members [88]. Some of the EMT-TFs promote resistance by enhancing drug efflux by ABC transporters. Overexpression of ATP-binding cassette C5 transporter (ABCC5) correlates with Forkhead box M1 (FOXM1) in paclitaxel-resistant nasopharyngeal carcinoma cells. Down-regulation of FOXM1 or ABCC5 reduces drug efflux and leads to cell death by paclitaxel [84].

### 3.4. Drug Activation and Inactivation

Anticancer drugs require metabolic activation, and thus cancer cells can develop resistance through drug inactivation or reduced drug activation. Drug activation and inactivation are seen in the glutathione S-transferase (GST) superfamily, a group of detoxifying enzymes that catalyze the conjugation of glutathione (GSH) to electrophilic compounds. GST enzymes induce drug resistance through direct detoxification of cancer drugs or by inhibiting the MAPK pathway [89]. Another mode of drug inactivation occurs through the cytochrome P450 (CYP) system. The CYP system consists of two classes. Class I includes enzymes CYP1A1, CYP1A2, CYP2E1, and CYP3A4, which are involved in the metabolism of drugs and procarcinogens. Class II consists of the enzymes CYP2B6, CYP2C9, CYP2C19, and CYP2D6, which are involved in drug metabolism. It is suggested that mutations in these enzymes would result in the breakdown and secretion of the drugs, thus reducing their optimum concentration leading to drug resistance [90,91]. CYP450 class I metabolizing enzymes are shown to inactivate the drug Irinotecan, a topoisomerase I inhibitor used for colon cancer treatment [92]. The DNA-binding glycopeptide drug bleomycin is inactivated by bleomycin hydrolase. Cancers resistant to bleomycin have high levels of this enzyme, whereas sensitive tumors (germ cell cancers, lymphomas, squamous carcinomas) have low levels [93].

### 3.5. Alterations in Drug Targets

Many anti-cancer drugs impose their cell cytotoxic properties via involving interactions with the intracellular target proteins. Any alteration in the expression level and/or function of these target enzymes eventually leads to the impaired function of drugs. Importantly, dysregulation of thymidylate synthase [1], dihydrofolate reductase [2,3] and topoisomerases I and II [4,5] are known to cause resistance against 5-fluorouracil and tomudex [6], methotrexate [7] and doxorubicin [8], respectively. A survival-threatening mutation in the BCR-ABL kinase drug binding site is a well-known risk factor that causes resistance to the drug Gleevec [94]. Besides, changes in the expression level of estrogen and progesterone receptors neutralize the impact of tamoxifen treatment in breast cancer patients [95].

### 3.6. Enhanced DNA Repair and Impaired Apoptosis

Inducing DNA damage is one of the modes of action of chemotherapeutic drugs to kill cancer cells. If the cells are able to repair the DNA damage caused, they will escape cell death and will develop chemoresistance. DNA damage response (DDR) mechanisms can reverse the drug-induced damage.

One of the DDR mechanisms carried out by the Wnt signaling pathway-induced O(6)-methylguanine DNA methyltransferase (MGMT) up-regulation conferred temozolomide resistance in an orthotopic murine model of glioblastoma multiforme. Inhibition of the Wnt pathway led to down-regulation of MGMT expression and restored the chemosensitivity to DNA-alkylating drugs. Similarly, the low expression of MRE11 positively correlated with a good response to chemotherapy and surgical resection after down-staging by chemotherapy [96]. Inhibiting DNA repair kinases prevented doxorubicin resistance in breast cancer cells [97]. Moreover, abnormal DNA repair activity was found in palbociclib (CDK4/6 inhibitor)-resistant breast cancer cells, whereas PARP inhibitors, olaparib and niraparib treatment could significantly inhibit palbociclib resistance in cancer cells [98,99]. Collectively, suppressing the DNA repair in the tumor cells could effectively overcome the chemoresistance, which needs to be studied further potentially via targeting the DNA repair genes specifically in the tumor cells.

The main goal of cancer treatment is cell death mediated by apoptosis. In recent times, many cancers have developed mechanisms to overcome chemotherapy-induced apoptosis. Apoptosis occurs through two pathways: an intrinsic pathway mediated by the mitochondria that involves B-cell lymphoma 2 (BCL-2) family proteins, caspase-9 and Akt, and an extrinsic pathway that involves ligands and cell death receptors such as FAS, TNF-R, linker proteins, and caspases-3, -6, -7 and -8. The up-regulation of the anti-apoptotic genes (such as BCL-2 and Akt) and down-regulation of pro-apoptotic genes (like BCL-2 associated X (Bax) and B-cell lymphoma-extra large BCL-XL) in tumor cells are associated with increased resistance to chemotherapy. Specifically, BCL-2/BCL-XL up-regulation is clearly associated with a poor prognosis in cancer. The ability of these proteins to antagonize the pro-apoptotic family of proteins such as Bax and Bak has been the key mechanism by which these cells acquire resistance to apoptosis [100]. High expression of BCL-2/BCL-XL and overexpression of caspase-3 were found to inhibit apoptosis in multiple myeloma and resulted in resistance of the cancer cells to bortezomib [101].

Collectively, a diverse set of mechanisms could result in resistance to the chemotherapeutic agents. We have covered these mechanisms separately, and they are briefly shown in Table 2. However, we also expect the reader to conceive the significance of the interplay between these mechanisms.

## 4. Strategies to Combat Chemoresistance in Cancer

**Figure 5:**
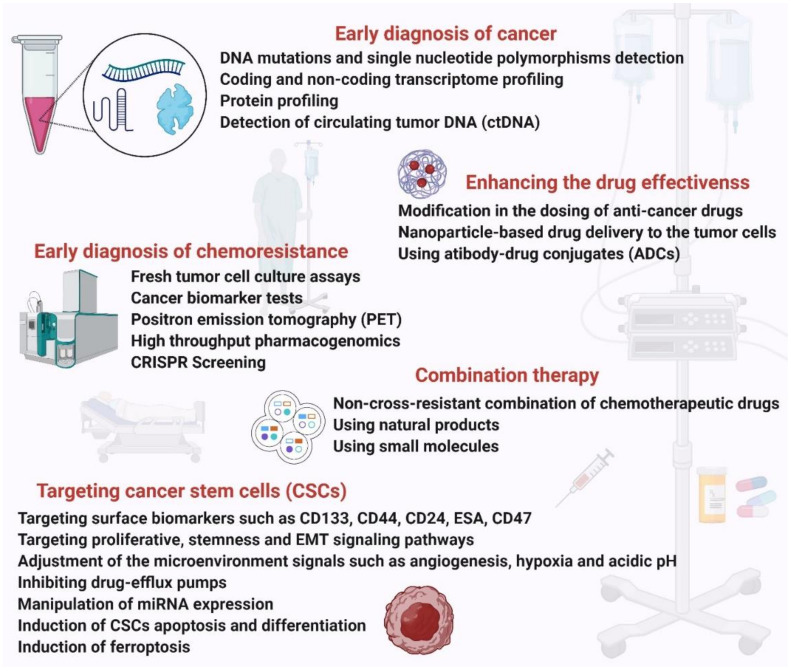
The strategies to combat chemoresistance in human cancers.

Chemotherapy is the standard approach for the treatment of cancer. However, the resistance of cancer cells to chemotherapeutic drugs reduces the efficacy of treatment and leads to poor survival in cancer patients. The key to combating chemoresistance is understanding the mechanisms underlying it and designing strategies to overcome them (Figure 5).

### 4.1. Early Diagnosis of Chemoresistance

Diagnosis of chemoresistance at the earliest stage will help improve cancer treatment. With the development of laboratory techniques like cancer genomics, transcriptomics, cancer proteomics, metabolomics and different tests, it is now possible to identify genes, markers and major components that contribute to drug resistance at different stages of tumorigenesis. Fresh tumor cell culture assays, cancer biomarker tests, and positron emission tomography (PET) tests are being performed to predict and diagnose chemoresistance in vitro and in vivo. These assays are currently being used to study tumor cell responses to drugs in several cancers like ovarian, lung, breast and cervical [108].

High-throughput pharmacogenomics and CRISPR screens are being used to investigate populations of cancer cells carrying sensitivity biomarkers and unexpectedly resistant (UNRES) cell lines for unique genetic alterations that may drive resistance. In one study, genomics of drug sensitivity in cancer (GDSC) and clinical trials reporting program (CTRP) datasets were analyzed to find UNRES cases and identify putative resistance biomarkers. Interrogating the foundations of drug resistance with publicly available CRISPR phenotypic assays assists in ranking resistance drivers and offering hypotheses for drug combinations [109]. The *EGFR*^T790M^ mutation and *PTEN* loss in lung adenocarcinoma cells treated with EGFR inhibitors have been proposed as the resistance biomarkers based on their hypothesis.

### 4.2. Enhancing the Drug Response Efficacy

Cancer drugs are usually administered to patients at a high dosage. However, recent studies from animals and theoretical models have shown that discontinuous dosing and modifying drug concentrations can combat drug resistance and improve patient survival. This approach was also found to be less toxic to cancer patients [110]. For example, discontinuous dosing of anaplastic lymphoma kinase (ALK) kinase inhibitors crizotinib and ceritinib may prolong control of ALK^+^ tumors [111]. This indicates that modification in the dosing of anti-cancer drugs could enhance the effectiveness of chemotherapy.

Dosing is not the only determinant for the ceiling of drug efficacy, but rather the toxicity could play a more important role. Nanomedicine [112], the use of nanostructured materials to serve as vehicles, has been emerging to not only boost the targeted delivery but also to alleviate the cytotoxicity of the chemotherapeutic reagents [113]. Although benefiting from nanoparticle platforms, such as liposomes, having overcome the drug resistance in hematological and germ cell cancers, the effectiveness of these nanocarriers has been unsuccessful in solid tumors [114]. More advancements in the use of nanocarriers remain to be developed in order to combat chemoresistance by means of nanoparticles. In parallel with nanoparticles, antibody–drug conjugates (ADCs) have been utilized to deliver the chemotherapeutic reagent specifically to the tumor cells. In this method, an engineered antibody with a very high affinity to a tumor cell antigen is bound to the cytotoxic reagents via a synthetic linker. This could directly deliver the chemotherapeutic reagent to the tumor cell and thereby reduce the cytotoxicity to the normal cells [115,116].

### 4.3. Use of Natural Products

Natural products with their diverse chemical structures and pharmacological benefits can serve as substrates to treat drug resistance. Natural products do so by two approaches. The first is they reduce drug efflux and thus maintain optimum concentration of the drug. The P-gp transporter protein is responsible for efflux of cancer drugs. Binding of P-gp to the drug results in activation of its ATP-binding domain and hydrolysis of ATP causing a change in the shape of P-pg, leading to drug efflux. Natural products that can inhibit the action of the P-gp transport system are being developed. For example, tanshinone microemulsion can significantly reverse drug resistance of K562/ADM cells by inhibiting the P-gp efflux pump effect and increasing the intracellular concentration of chemotherapeutic drugs. Other natural products effective in combating drug resistance include tetrandrine, quercetin, grape-seed polyphenols, and tea polyphenol [117].

The second approach is they induce non-apoptotic cell death in cancer cells. Natural products induce non-apoptotic cell death processes like necroptosis (Shikonin and its analogs, MAM), autophagy (Arsenic trioxide, G. lucidum triterpene, resveratrol, oridonin, allicin), methuosis (chalcone; ginsenoside; curcumin; quercetin) and oncosis (sanguinarine, solamargine, artesunate, rosin) in cancer cells overcoming chemoresistance [118].

Fourteen single compounds shown to be able to overcome cancer cell drug resistance are evodiamine, peiminine, isorhynchophylline, berberine, ephedrine, ginsenoside Rb1, oridonin, oxymatrine, methylether-scutellarein, sodium norcantharidate, phenyl-propanoid glycoside, retinoic acid, schizandrin A, and baicalin [117]. There exists an inverse correlation between consumption of dietary polyphenols and the risk of cancer. Polyphenols possess antioxidant capacity and inhibit activation of procarcinogens, cancer cell proliferation, metastasis, angiogenesis and drug efflux transporters. They also induce apoptosis in cancer cells and modulate immune responses and inflammatory cascades [119].

Natural products such as Vitamin C, curcumin and its derivatives, flavones and isoflavones, naphthoquinones, anthrocyclins are found to aid in overcoming cisplatin chemoresistance in bladder cancer and enhance the efficacy of cisplatin while reducing or blocking its toxic effects [120].

### 4.4. Targeting CSCs

CSCs are a sub-population of cells in cancer that possess the ability to self-renew, initiate tumor formation, metastasis, EMT and also cause chemoresistance and cancer relapse. CSCs have been identified in breast, brain, thyroid, melanoma, colon, pancreatic, liver, prostate, lung, head and neck, ovarian, and stomach cancers. CSCs are known to cause chemo- and MDR through their responses such as immune evasion, drug efflux by overexpression of ABC transporters, EMT, increased DNA repair, etc. Thus, targeting this sub-population of cells will serve as a promising strategy to overcome chemoresistance and improve clinical outcomes.

The common approaches to targeting CSCs are targeting their surface biomarkers such as CD133, CD44, CD24, ESA, CD47, targeting proliferative, stemness and EMT signaling pathways like Notch, Hedgehog, P1_3_K/Akt, Wnt, and NF_k_B that regulate CSC self-renewal and differentiation [121], adjustment of the tumor microenvironment signals such as angiogenesis, hypoxia and acidic pH, inhibiting drug-efflux pumps, manipulation of miRNA expression, induction of CSCs apoptosis and differentiation, and induction of ferroptosis [122,123,124].

Some examples of these strategies are that aberrant activation of the Wnt/β-catenin pathway in CSCs was closely associated with tumorigenesis in many tissues. An antibody specific to frizzled7, a Wnt receptor, depleted clonogenicity and tumorigenicity in tumors. Dickkopf-1 (Dkk1), a major secreted Wnt signaling antagonist, bound to the low-density lipoprotein receptor-related protein-6 (LRP6), an essential co-receptor for canonical Wnt signaling. Recently, salinomycin, an antibiotic potassium ionophore, has been reported to inhibit breast CSCs and target the Wnt pathway by blocking the phosphorylation of LRP6 [125].

Metformin (1, 1-dimethyl biguanide), a widely used anti-hyperglycemic agent, sensitizes tumor response to various chemotherapeutic drugs. Metformin selectively targets CSCs and improves the hypoxic microenvironment, suppresses tumor metastasis and inflammation, as well as regulates metabolic programming, induces apoptosis, and reverses epithelial–mesenchymal transition and MDR in breast cancer [44].

### 4.5. Combination Therapy

Another strategy to overcome chemoresistance in cancer is the use of multiple anticancer agents at an optimum synergistic ratio. Monotherapy reinforces alternative molecular pathways in cancer cells, leading to chemo-resistant mutations and cancer relapse. The multiple anticancer drugs are often administered using a nanocarrier that further increases their therapeutic effect. Nanocarriers overcome the limitations of standard combination therapy as they deliver several drugs to the same tumor cell in one package, they promote their synergistic action, they can deliver a high drug dose which may overwhelm drug efflux mechanisms of cancer cells and they show enhanced accumulation, permeability and retention at the tumor site. Preclinical studies have shown that multidrug-loaded nanocarriers can reverse drug resistance more efficiently than conventional combination therapies [126].

In a recent study, resveratrol and paclitaxel were co-delivered to drug-resistant breast tumors using a PEGylated liposome therapy. The composite liposome generated potent cytotoxicity against the drug-resistant MCF-7/Adr tumor cells in vitro and enhanced the bioavailability and the tumor-retention of the drugs in vivo. Moreover, systemic therapy with the composite liposome significantly inhibited drug-resistant tumors in mice, without an increase in toxicity. These results suggested that the co-delivery of resveratrol and a cytotoxic agent in a nanocarrier may potentially improve the treatment of drug-resistant tumors [127].

Planetary ball milled (PBM) nanoparticles encapsulated with resveratrol, and in combination with docetaxel and conjugated with folic acid (FA) on the surface were co-delivered to prostate cancer cells. This combination therapy resulted in down-regulation of anti-apoptotic genes, increased cytotoxicity of the drugs as well as a decrease in expression of drug efflux transporter proteins, reversing drug resistance in prostate cancer [128].

### 4.6. Use of Inhibitors

Small molecules that are low-molecular-weight organic compounds with the ability to regulate biological processes have been emerging as effective and robust substances to improve cancer treatment via overcoming the chemoresistance phenomenon. These nanosized molecules have shown a high therapeutic index and improved outcomes upon using either the most suitable inhibitor for a particular cancer or the combinational mixture of them. Osimertinib, which is an EGFR-mutant-selective inhibitor, and alectinib, which is an ALK inhibitor have been displayed to elevate the survival in non-small-cell lung cancer [129,130]. Compelling evidence supports the notion that using small molecules before the observation of resistance and in combination with other chemotherapeutic reagents could prolong the survival of cancer patients. For instance, olaparib resulted in prolonged survival in ovarian cancer patients who received a platinum-based chemotherapy regimen as compared with the patients who received platinum-based chemotherapy alone [131], suggesting that the early and aggressive combinational treatment may have a deeper impact on the prognosis of patients.

## 5. Conclusions and Future Perspectives

Chemoresistance remains a lethal challenge in the realm of cancer biology and clinics. Various determinants with their modes of action have been reported with clinical implications. However, many patients regrettably die due to chemoresistance-induced failure in treatment. This failure is mediated by the tumor-specific characteristics in each patient, which eventually dictates the resistance and cancer progression in unpredictable and yet to be known mechanisms. Therefore, the resistance problem has remained an unachievable target.

It is a universal notion that larger tumor size correlates with worse prognosis, thereby they are inversely associated with curability. This highlights the tumor size as a key determinant of therapy success in clinics. There are a number of hypotheses to deeply determine the role of tumor size in the treatment strategies. Log-kill hypothesis states that combining multidrug chemotherapy that kills the tumor cells logarithmically over multiple cycles could eradicate cancer [132]. This model explains well only some cancer types like lymphoma and germ cell tumors. Thus, it is not consistent with all cancer types. A more accurate model, the Goldie–Coldman hypothesis, accounts for tumor size with the incorporation of the drug resistance phenomenon [133]. Based on this model, bigger tumors have a higher rate of mutation, and thus a higher number of drug-resistant clones. Due to the presence of various clones with different drug-resistance mechanisms in a particular tumor, using a multidrug regimen at the same time (Log-kill model) would not be effective. The emergence of using lower doses of drugs in multidrug therapy is an extra adverse affecting factor. The Goldie–Coldman model, therefore, suggests that alternating the non-cross-resistant combination of chemotherapeutic drugs, with higher doses, could improve the effectiveness of treatment. However, this model has not been explored in pre-clinical or clinical trials, reflecting that complicated concerns have been considered for such a hypothesis [134].

**Figure 6:**
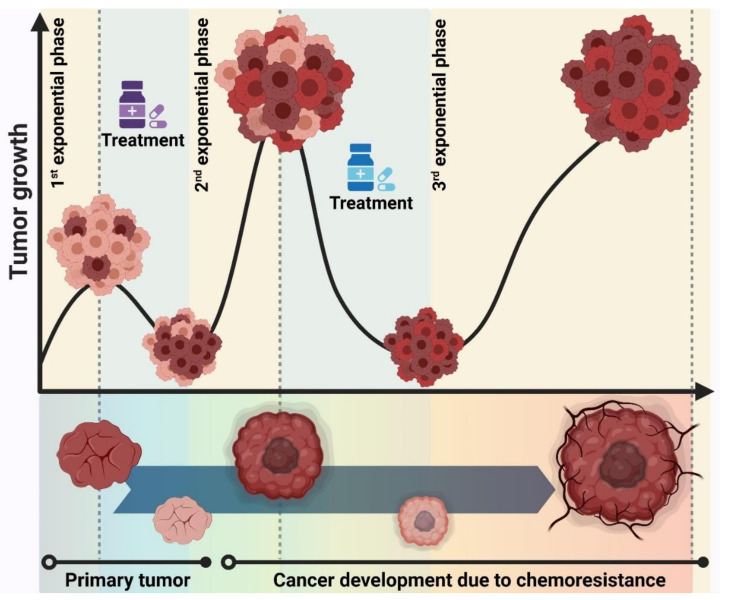
The Norton–Simon hypothesis describes the logical relationship between the tumor growth rate and chemoresistance phenomenon in cancer development.

The Norton–Simon hypothesis, which is the most comprehensive model, states that tumor growth has two phases [135]. When the tumor size is small, the tumor cells grow exponentially (aggressive cells) to faster form a larger tumor size. This tumor then enters the plateau phase where the cells grow slower (indolent cells) [136]. The key determinant in cancer curability is therefore the effect of the drug on the tumor size. Upon the administration of chemotherapy on a larger tumor, the tumor size rapidly shrinks due to the indolent cell death while this leads to the enrichment of drug-resistant cells. The consequence of the therapy is thereby a much smaller tumor in size but enriched in drug-resistant cells. The enriched tumor cells then start a new exponential growth rate, resulting in the formation of another larger tumor which is now very resistant to chemotherapeutic agents (Figure 6). The Norton–Simon hypothesis thus suggests that the success rate of therapy depends on how the treatment strategy could prevent exponential cell growth after the first chemotherapy dose or between different doses. The proposed treatment strategy could be administrating the most effective dose level of a drug over short time intervals to maintain the shrinkage of the tumor, which is called the dose-density model. This model has shown clinical proof of concept in breast and ovarian cancer with positive effects on the survival of patients [137,138,139]. However, this model has not been able to convert an ineffective therapy to the effective one. Incorporating the targeted therapy, together with chemotherapy, into the dose-density model could help improve the success rate of cancer curability for a wider range of human cancers.

Collectively, this seems quite logical that early diagnosis of cancer could help us prevent the first exponential growth phase, thereby minimizing the accumulation of intrinsic drug-resistant cells in the larger tumor size. This could massively influence the curability of cancer. Various methods are being utilized to detect cancer in pre-symptomatic steps. For example, mammography for breast cancer and prostate-specific antigen (PSA) screening for prostate cancer. These methods, however, show low sensitivity and specificity and have excessive false-positive results [140,141,142]. Complementary approaches by means of molecular tools such as screening the DNA mutations [143,144], single nucleotide polymorphisms [145,146,147], expression profiling of coding [148,149] and non-coding genes [54,150,151,152], and detecting the circulating tumor DNA (ctDNA) [153,154,155] have been improving the early diagnosis of a wide range of human cancers.

Through early diagnosis, which means lower heterogeneity, and preventing the exponential growth of cancer cells, dose-density therapy with short intervals using either multidrug chemotherapy or alternating chemotherapy regimens could reduce or optimistically eradicate both intrinsic and acquired drug-resistant cells. Focusing on both early detection and more effective treatment could eventually lead to the uprooting of tumor cells, which correlates with the elongated survival of cancer patients.

## Figures and Tables

**Table 1:** Overexpressed ABC proteins affecting the efflux of chemotherapeutic drugs in human cancers.

ABC Protein	Overexpressed in Cancers	Drugs Effluxed
P-glycoprotein (P-gp, ABCB1)	Lung, liver, kidney, rectum, colon, leukemias, myeloma, breast, ovary	Doxorubicin, epipodophyllotoxins, anthracyclines, vinca alkaloids, bisantrene, colchicine, taxanes, imatinib, saquinavir, camptothecins, thiopurines, actinomycin D, methotrexate, and mitoxantrone, paclitaxel, docetaxel
Breast cancer resistance protein (BCRP/ABCG2)	Small cell lung cancer, breast, prostate, esophageal, leukemia, colon, stomach	Cisplatin, doxorubicin, etoposide, Mitoxantrone, topotecan, anthracyclines, irinotecan, methotrexate, paclitaxel, TKI
Multidrug resistance-related proteins (MRP1/ABCC1 and MRP2/ABCC2)	Neuroblastoma, lung, breast, esophageal, leukemia	etoposide, methotrexate, doxorubicin, epirubicin and vincristine, anthracyclines, etoposide, camptothecins, methotrexate, mitoxantrone, vincristine, vinblastine, irinotecan, TKI as imatinib

ABCB1: ATP-binding cassette B1 transporter; BCRP: Breast cancer resistance protein; ABCG2: ATP-binding cassette G2 Transporter (Junior blood group); MRP2: Multidrug resistance protein 2; ABCC1: ATP-binding cassette C1 transporter; TKI: Tyrosine kinase inhibitor; ABCC2: ATP-binding cassette C2 transporter.

**Table 2:** The underlying mechanisms causing chemoresistance in cancer.

Mechanims	Target	Anti-Cancer Drugs	Cancer Type
Drug efflux	ABC transporters	Doxorubicin, epipodophyllotoxins, anthracyclines, vinca alkaloids, bisantrene, colchicine, taxanes, imatinib, saquinavir, camptothecins, thiopurines, actinomycin D, methotrexate, and mitoxantrone, paclitaxel, docetaxel	Most cancers [64,65,66,67,68,85]
Drug influx	SLC transporters	Pralatrexate and methotrexate	ALL, multiple myeloma, Osteosarcoma [62,63,79]
Drug influx	pH	Daunorubicin, doxorubicin, mitoxantrone, cyclophosphamide, chlorambucil, cisplatin	Most cancers [32]
Tumor microenvironment	Hypoxia	Topoisomerase-ll targeted drugs (idarubicin, daunorubicin, and doxorubicin)	Solid tumors [102]
Enhanced DNA repair	MGMT	Temozolomide	Various cancers [103]
	Wip1, a suppressor of the ATM-dependent signaling pathway	Cisplatin	Gastric cancer [104], Oral squamous cell carcinoma [96]
EMT	Snail and twist	Cyclophosphamide, gemcitabine	Colon, pancreatic [105,106]
Signaling pathways	WNT/β-Catenin	Cisplatin, doxorubicin, 5-fluorouracil, paclitaxel	Hepatocellular carcinoma, Neuroblastoma, ovarian and colon cancers, glioma [107]
	Notch	Oxaliplatin, cisplatin, temozolomide	

EMT: Epithelial-mesenchymal transition; ABC: ATP-binding cassette; SLC: Solute carrier; MGMT: O(6)-methylguanine DNA methyltransferase; ATM: Ataxia telangiectasia mutated; ALL: Acute lymphoblastic leukemia.

## Data Availability

Not applicable.

## Abbreviations

ABC	ATP-binding cassette
ABCB1	ATP-binding cassette B1 Transporter
ABCC1	ATP-binding cassette C1 Transporter
ABCC2	ATP-binding cassette C2 transporter
ABCC5	ATP-binding cassette C5 Transporter
ABCG2	ATP-binding cassette G2 Transporter (Junior blood group)
ADC	Antibody-drug conjugate
Akt	Protein kinase B
ALK	Anaplastic Lymphoma Kinase
ALL	Acute lymphoblastic leukemia
ATM	Ataxia telangiectasia mutated
BAX	BCL2 associated X
BCL-2	B-cell lymphoma 2
BCL-XL	B-cell lymphoma-extra large
BCRP	Breast cancer resistance protein
BCSCs	Breast cancer stem cells
CSCs	Cancer stem cells
ctDNA	circulating tumor DNA
CTRP	Clinical trials reporting program
Cul3	Cullin3
CYP	Cytochrome P450
DDR	DNA damage response
DKK1	Dickkopf-1
EMT	Epithelial-mesenchymal transition
EMT-TFs	EMT-inducing transcriptional factors
FA	Folic acid
FOXC2	Forkhead box C2
FOXM1	Forkhead box M1
GDSC	Genomics of drug sensitivity in cancer
GST	Glutathione S-transferase
HCC	Hepatocellular carcinoma cells
ICAM-1	Intercellular adhesion molecule 1
KEAP1	Kelch-like ECH-associated protein 1
LFA-1	Leukocyte function-associated antigen 1
LncRNA	Long non-coding rnas
LRP	Lung resistance-related protein
LSCC	Lung squamous cell carcinoma
LUAD	Lung adenocarcinoma
MAPK	Mitogen-activated protein kinase
MDR	Multidrug resistance
MGMT	Methylguanine DNA methyltransferase
MRE11	Double strand break protein
MRP1	Multidrug resistance-associated protein 1
MRP2	Multidrug resistance protein 2
NFE2L2	Nuclear factor (erythroid-derived 2)-like 2
NF_k_B	Nuclear factor-κB
NSCLC	Non-small cell lung cancer
OS	Overall survival
PARP	Poly(ADP-Ribose) polymerase 1
PBM	Planetary ball milled
PET	Positron emission tomography
P-gp	P-glycoprotein
PI_3_K	Phosphoinositide 3-kinase
PSA	Prostate-specific antigen
RFC	Reduced folate carrier
TGF-β	Transforming growth factor β
TKI	Tyrosine kinase inhibitor
TME	Tumor microenvironment
Topo-II	Topoisomerase II
TTF	Time to treatment failure
Tβ4	Thymosin β4
UCA1	Urothelial cancer associated 1
UNRES	Unexpectedly resistant
VCAM	vascular adhesion molecule
VEGFR	Vascular endothelial growth factor receptor
VLA-4	Very late antigen 4
Wnt6	Wnt family member 6
ZEB1	Zinc finger e-box binding homeobox 1
